# Systems Approaches to Animal Disease Surveillance and Resource Allocation: Methodological Frameworks for Behavioral Analysis

**DOI:** 10.1371/journal.pone.0082019

**Published:** 2013-11-29

**Authors:** Karl M. Rich, Matthew J. Denwood, Alistair W. Stott, Dominic J. Mellor, Stuart W. J. Reid, George J. Gunn

**Affiliations:** 1 Department of International Economics, Norwegian Institute of International Affairs (NUPI), Oslo, Norway; 2 School of Veterinary Medicine, College of Medical, Veterinary, and Life Sciences, University of Glasgow, Glasgow, Scotland; 3 Future Farming Systems, Scotland’s Rural College (SRUC), Edinburgh, Scotland; 4 School of Veterinary Medicine, College of Medical, Veterinary, and Life Sciences, University of Glasgow, Glasgow, Scotland; 5 Royal Veterinary College, North Mymms, Hatfield, Hertfordshire, England; 6 Future Farming Systems, Scotland’s Rural College (SRUC), Drummondhill, Inverness, Scotland; The Pirbright Institute, United Kingdom

## Abstract

While demands for animal disease surveillance systems are growing, there has been little applied research that has examined the interactions between resource allocation, cost-effectiveness, and behavioral considerations of actors throughout the livestock supply chain in a surveillance system context. These interactions are important as feedbacks between surveillance decisions and disease evolution may be modulated by their contextual drivers, influencing the cost-effectiveness of a given surveillance system. This paper identifies a number of key behavioral aspects involved in animal health surveillance systems and reviews some novel methodologies for their analysis. A generic framework for analysis is discussed, with exemplar results provided to demonstrate the utility of such an approach in guiding better disease control and surveillance decisions.

## Introduction

Demands for animal disease surveillance systems have increased in recent years, given the changing landscape of global trade and increased concerns about exotic pathogens [1]. Various rubrics of surveillance modalities, ranging from targeted surveillance [2], risk-based surveillance [1], and participatory disease surveillance [3] have emerged in response as a way of making such programs more cost-effective; recent research has synthesized these terminologies [4]. Much of the focus in the veterinary epidemiology literature, on risk-based surveillance in particular, has been on technical criteria associated with risk and risk factors that underpin disease, and the means by which disease searching and monitoring better account for such criteria [1]. 

By contrast, analyses that focus on the resource allocation side of the surveillance equation are much more limited. Recently, Cannon [5] reviewed different metrics of surveillance resource optimization problems based on different objectives by decision-makers (e.g., maximizing detection, minimizing detection time, maximizing benefits from early detection), providing generic examples for setting up each approach. Such optimization approaches to surveillance have been applied in other related areas including fisheries surveillance [6] and invasive species management in the ecology literature [7-9], but have not generally been utilized in the veterinary literature. Rather, surveillance programs are generally analyzed in more broad terms, such as a component of a simulation analysis of disease mitigation options [10-15] or in benefit-cost analyses using economic welfare indicators (producer and consumer surplus) [16,17]. Häsler et al. [18,19] combined simulation tools on the benefits side with an accounting of various mitigation costs to compute the net margin available for surveillance costs in the context of bluetongue and bovine viral diarrhea (BVD), respectively. Recently, Howe, Häsler, and Stärk [20] provided a theoretical exposition on the relationship between surveillance and intervention expenditures. Rather than focusing solely on the tradeoffs between surveillance and intervention, the authors highlighted the need to jointly consider both types of expenditures together to maximize the net benefits associated with avoided disease losses.

In the context of risk-based surveillance, Prattley et al. [21] applied portfolio theory from the finance literature to develop numerical indicators that provide guidance on the allocation of surveillance resources in light of their risk factors. Earlier work by Mariner et al. [22] also utilized a number of performance indicators to evaluate surveillance programs associated with Rinderpest control, which Benschop et al. [23] applied a risk analysis framework to highlight specific spatial risks associated with herd sizes, feeding practices, and health status that are attributable to disease prevalence. Gilbert, Häsler, and Rushton [24] recently developed survey-based protocols to probabilistically assess various drivers and predictors associated with farm and veterinary behavior that influence reporting and compliance. Farm-level predictors included characteristics such as the production system employed, monitoring scheme membership, frequency of veterinary contact, herd size, and farm-level record keeping. However, none of these risk-based approaches looked explicitly at how best to allocate resources given different risk factors. 

An additional, and important, gap in all of these analyses is couching the optimal allocation of resources (however defined), with the risk factors that might significantly modulate their effectiveness in mitigating disease itself. Hauser and McCarthy [8] come closest in this regard by paying particular attention to the spatial allocation of resources based on the efficacy and presence of disease over a landscape. Despite this, their analysis was static, and did not link the allocation and efficacy of surveillance resources to subsequent disease incidence in future periods. However, in an animal disease setting, where the behavior of agents can actively influence the epidemiology of disease [25], it is critical to evaluate resource allocation in its appropriate dynamic systems setting. Put differently, decisions by policymakers in surveillance programs will have an effect on the behavior of producers and other actors in the agri-food chain, which in turn can influence the epidemiology of disease, and consequently, the cost-effectiveness of surveillance systems over time. These feedbacks can be complex and not intuitive, requiring more nuanced approaches to their economic analysis. 

Related to this, and a crucial factor which has rarely been considered, is that the nature of surveillance itself is not static. Surveillance programs will differ in nature not only based on the context of the disease, but also on the efficacy of disease control programs and the objectives and goals of decision makers themselves. While Hadorn and Stärk [26] recently contrasted the effectiveness of active and passive surveillance systems in a decision-tree framework, their analysis did not capture how these objectives could change over time as the success in controlling (or failing to control) disease changes. By contrast, Häsler et al. [27] considered surveillance in the context of the type of disease and the evolutionary stages of diseases over time. They considered three stages of disease: (i) sustainment, in which the surveillance objective is either to maintain disease freedom or detect disease; (ii) investigation, in which the objective is to obtain more information about an endemic or epidemic disease; and (iii) implementation, in which surveillance serves as an information source for mitigation options. Such a conceptual framework highlights the dynamism inherent within surveillance programs and strongly suggests a need for system-based empirical approaches to address them.

In this paper, we provide a more robust conceptual framework for the allocation and composition of surveillance resources, overlaying the socio-economic drivers of risk and disease response alongside the biological and spatial dimensions of disease. In this manner, the paper builds on two recent analyses that examined resource allocation issues in a disease setting. First, it extends the work of Cannon [5] by adding the systems dimension regarding the behavioral aspects, attitudes, constraints, and practices of producers and other recipients of surveillance resources [24]. The approach is akin to the recent analysis of Duintjer Tebbens and Thompson [28] that analyzed alternative decision rules for resource allocation in its dynamic epidemiological context. However, our approach highlights the interface of the disease epidemiology with different types of actors in the agri-food chain based on their risk profiles [25], while maintaining the alternative decision rule metrics of past analyses.

An advantage of this framework is that it can accommodate significant heterogeneity and feedback mechanisms in this socio-economic overlay, based on data availability and the nature of the disease in question. The analysis presented here is necessarily dynamic, and has the advantage of modeling both the evolution of surveillance resources based on their effectiveness over time and taking into account external drivers that might influence uptake, thus providing a set of empirical tools that combine and overlay the framework found in Häsler et al. [27] and risk factors revealed in Gilbert, Häsler, and Rushton [24]. Our analysis first presents a series of general principles that underpin this approach, followed by a generic example of an application of these methods to disease control in Scotland. 

## Materials and Methods

### 1: Conceptual framework: principles for analysis

**Figure 1:**
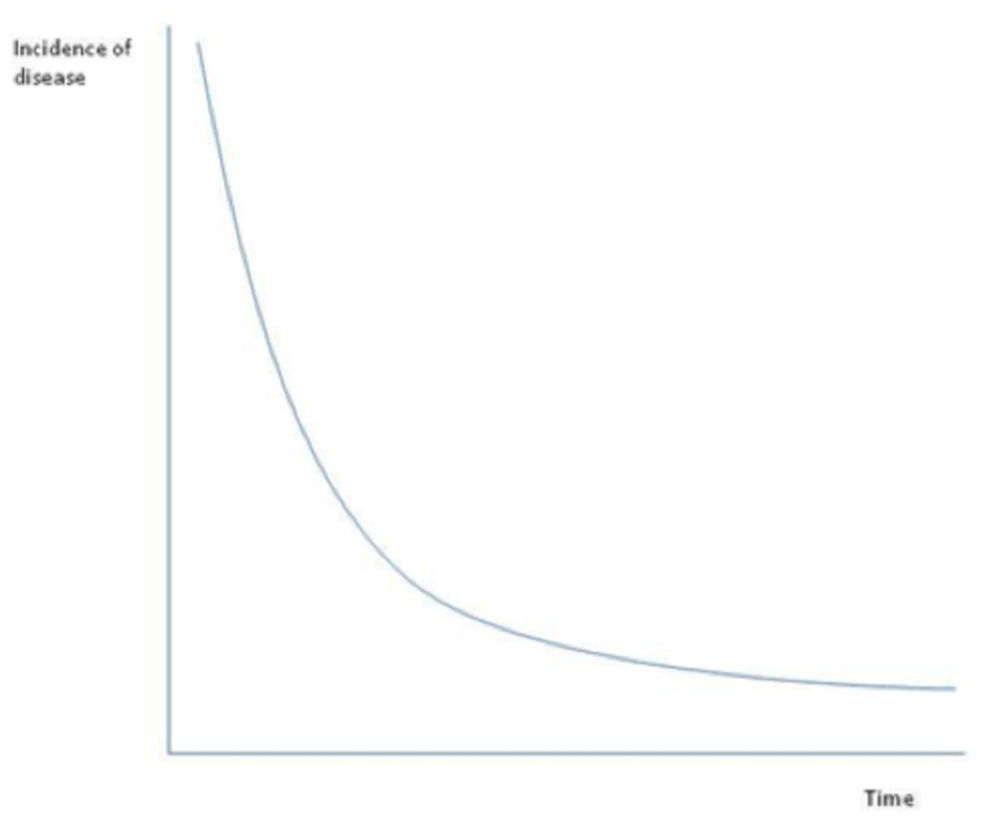
Decay curve relating disease incidence with surveillance efforts over time.

**Figure 2:**
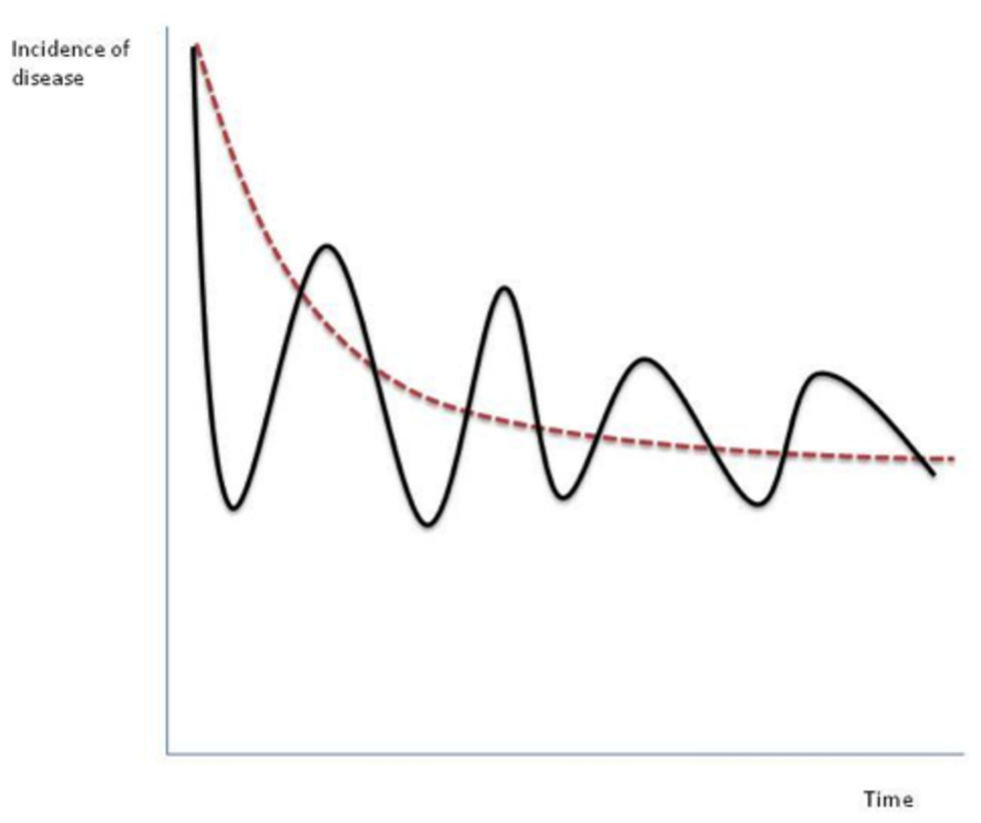
Alternative paths of disease incidence over time.

A starting point for our analysis is to first place surveillance efforts in their epidemiological and socio-economic contexts. One way to conceptualize surveillance efforts is in their contribution to the reduction of disease. Figure 1 characterizes this relationship in terms of a “decay curve” of disease, whereby the incidence of disease is posited to (ideally) decline over time as surveillance resources are allocated towards the detection and mitigation of disease. The smooth shapes of such decay curves are more prominent in diseases in which wide-scale eradication programs are implemented, as illustrated in the examples of polio, smallpox, and malaria [28]. At different points along this decay curve, one will utilize different types (and mixes) of surveillance and mitigation strategies to bring disease to lower and lower levels. In endemic settings, the decay curve might level off at some non-zero level or oscillate in regular intervals on the basis of a variety of agro-ecological, climatic, socio-economic, or other factors (Figure 2).

This latter point is particularly salient in many disease instances, but also illustrates a fundamental point often overlooked in the literature; namely, surveillance activities do not take place in a vacuum. As different programs are put into place, they will have a direct influence on the epidemiology of the disease (through control measures), and an indirect effect on behaviors taken by recipients of surveillance services that will modulate (positively or negatively) those efforts. Meanwhile, other changes take place that are not directly associated with the decay curve or its surveillance programme but nevertheless have an important influence on them. These might include progressive changes in the agricultural industry e.g. those associated with the ‘treadmill of technology’ or sudden changes associated for example with policy intervention.

**Figure 3:**
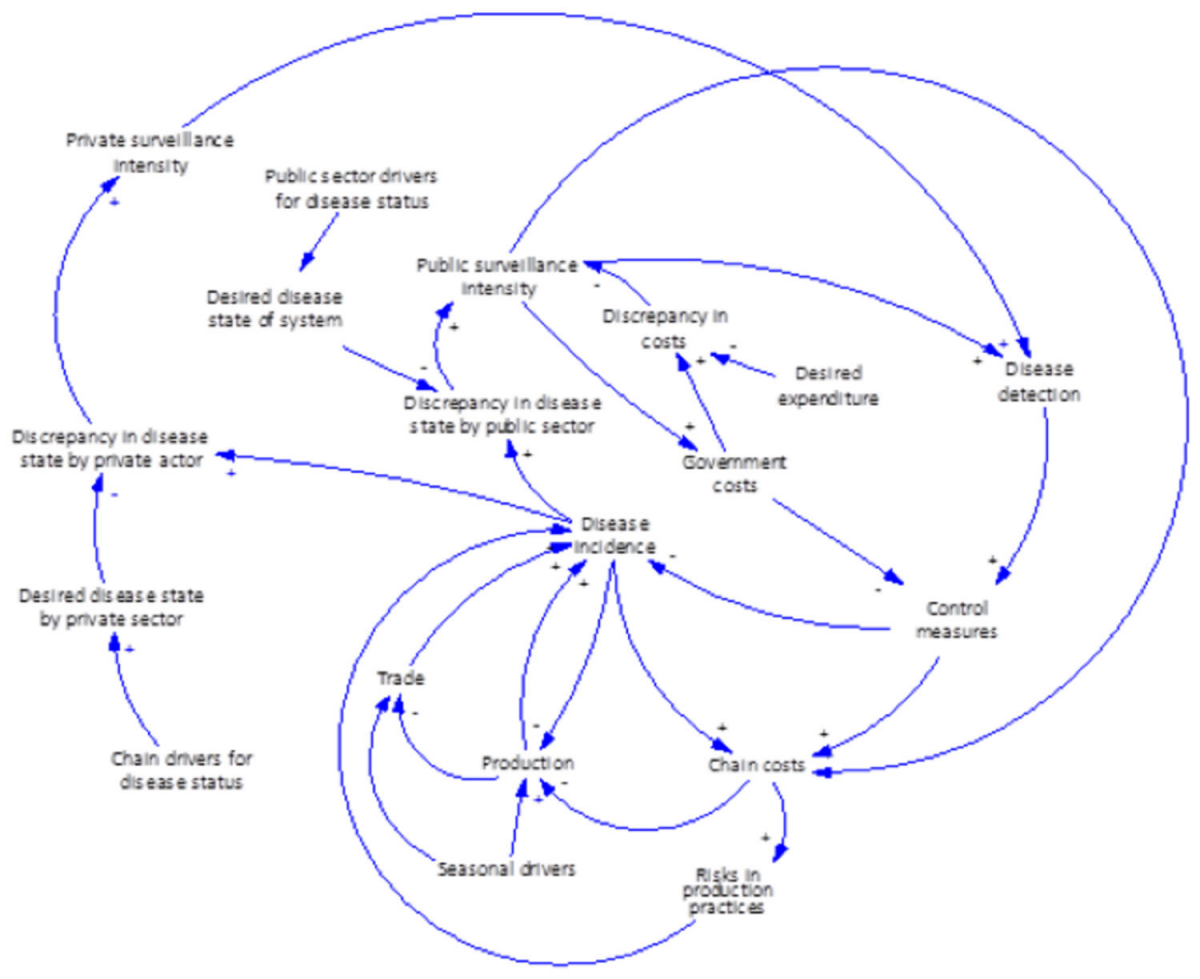
Causal loop diagram of surveillance in its systems setting.

Figure 3 illustrates these interactions in a causal loop diagram, a commonly used tool in the system dynamics literature to illustrate the feedback mechanisms present in complex systems [29]. Note that Figure 3 is an abstraction of many of the behaviors implicit in this system and necessarily excludes a number of key influences to simplify the analysis. The inner loop of the diagram provides the standard conceptualization of the role of surveillance – greater intensity in disease surveillance leads to more detections, and correspondingly more control measures, which over time should have the effect of bringing disease down to its desired level (possibly zero). However, as the diagram also illustrates, both surveillance programs and control measures impose costs on producers (and others in the agri-food chain). This might directly influence the level of production (less animals produced), modulating the incidence of disease downward. On the other hand, it could also lead to more risky behaviors that increase the risk of disease and lead to more trade (to meet consumer demand) from areas with more or less disease risk. These risk factors can be further segmented based on the type of disease considered (endemic vs. exotic, diseases that affect international trade), geography, types of production systems, capacity and knowledge from producers, seasonal drivers, as well as the interaction between producers through trade or other social networks. The importance of various factors will necessarily be disease-specific, with some factors much more important than others depending on the disease. Countervailing this further are the pressures surveillance and control programs face from resource constraints on budgets, which place limits on the level of efforts that can be made from surveillance. An important aspect of Figure 3 is in directly illustrating the relationships between the disease, its socio-economic risk factors, and the role surveillance efforts play in influencing this feedback structure. Correspondingly, any optimal allocation of surveillance resources needs to account for these dynamic impacts as additional constraints. 

An important related issue further illustrated in Figure 3 involves distinguishing who carries out surveillance activities themselves i.e., whether they are managed by the public sector or carried out privately among different actors in the agri-food chain. These motivations might be rather different. From the standpoint of the public sector, regulatory mandates (including compensation schemes in place) might shape protocols for surveillance, while for the private sector, these may be driven by competitive factors, such as the need to differentiate products in the marketplace. Among private sector actors themselves, and in different agri-food chains (e.g., commercial-oriented production vs. hobby farms), these motivations might differ significantly. Furthermore, surveillance activities conducted by the private and public sectors may overlap.

### 2: A review of candidate methodologies for analyzing the systems setting of surveillance

There are a couple of potential methods that more explicitly model the complexity of the problem discussed above. Such methods move away from optimization approaches [5], though these methods can represent a first approximation to the complexity of the surveillance problem, albeit without feedback effects implicitly considered. One method of setting up this type of problem is to model the system illustrated in Figure 3 directly as a system dynamics (or SD) problem. In this manner, the allocation of surveillance and mitigation resources can follow various decision rules established within the model [28], as can various strategies for disease control itself [30]. In this manner, Homer and Hirsch [31] examined the tradeoffs between diagnostic and therapeutic interventions in a generic model of public health. As will be seen shortly, such an approach can be easily adapted in a model of animal disease surveillance. A further advantage of a systems approach is the ability to overlap relevant socio-economic drivers that might influence the allocation of resources themselves. For instance, Ulli-Beer et al. [32] incorporated socio-economic behavior and attitudes towards waste and recycling decisions in a model that sought to optimize government budget resources towards incentives for effective waste management. Rich [25] proposed a way to link economic decisions with biological drivers of disease, but cost implications of alternative strategies from the epidemiological side were not considered. The manner in which economic agents are modeled can take a number of forms, depending on data availability, level of analysis, production system, and spatial diversity.

**Figure 4:**
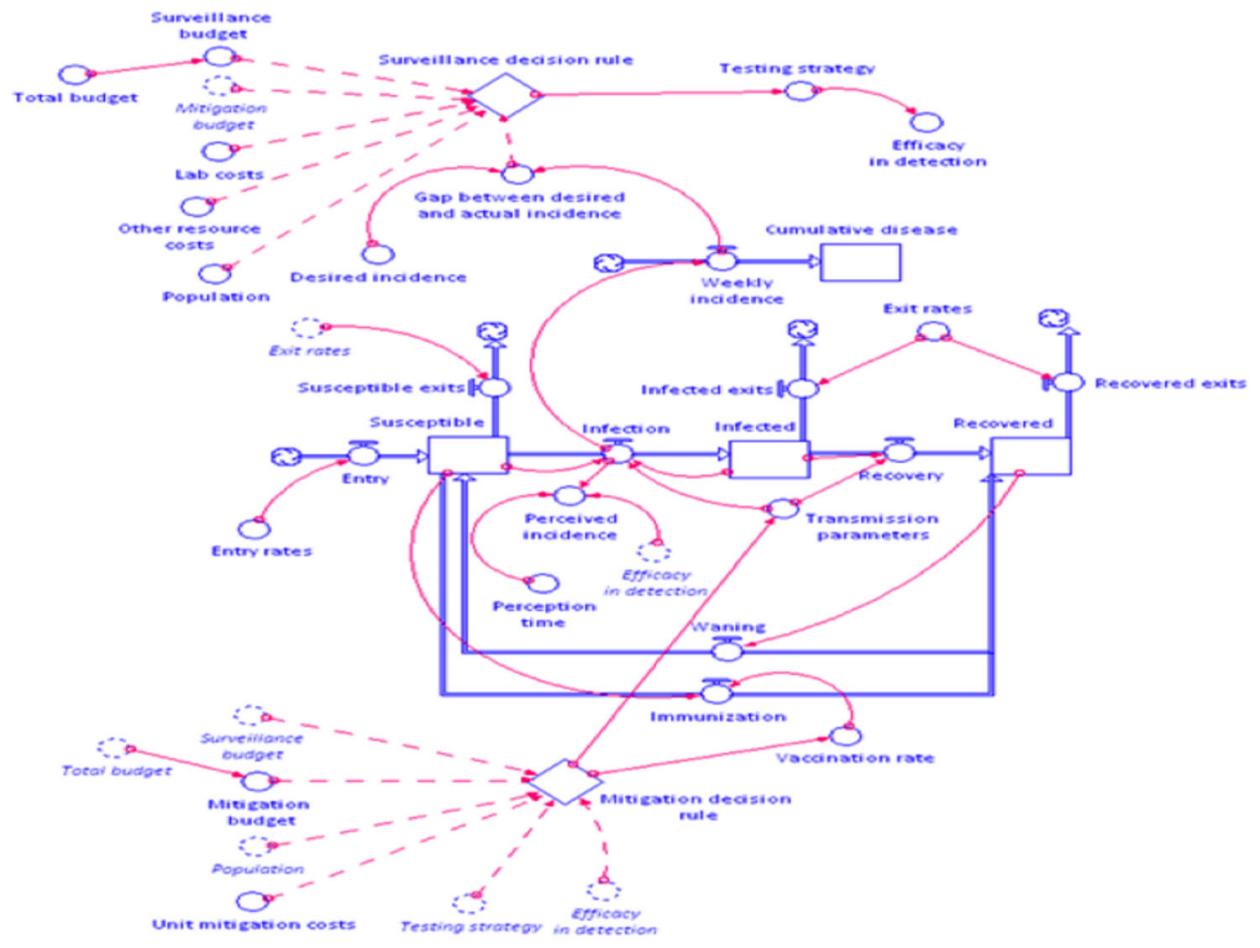
A system dynamics model of the interface between surveillance and disease spread.

Figure 4 provides a relatively generic template for incorporating surveillance and mitigation decisions into a disease control framework, extending the approach of Duintjer Tebbens and Thompson [28] and using the iThink software system (http://www.iseesystems.com). In this model, a simple S-I-R model of disease spread is developed that traces the evolution of animals or herds between different disease states of nature. In the diagram, the rectangles represent stocks of animals or herds at any given period of time, i.e., the number of animals/herds in the states susceptible, infected, or removed. The wide arrows denote the flows of animals/herds between different states. In the mathematical language of S-I-R models, these would be the differential equations that would underpin the movement of actors between states. The small circles and thin arrows that connect them to stocks, flows, or other circles are parameters that relate stocks and flows (and parameters). These can include rates of disease transmission, populations of animals/herds, efficacy rates of vaccination, and so on. In the model, transitions between susceptible and recovered (via vaccination) and recovered to susceptible (due to waning immunity) are included, while other disease states included latency and incubation periods could also be added [30]. A powerful advantage to modeling in iThink is the graphical representation of complex, non-linear systems of differential equations. Indeed, behind the graphical interface are functional forms that relate stocks, flows, and parameters in line with standard epidemiological theory.

At the top and bottom of Figure 4 are two diamond shapes that denote decision processes. These decision processes take as inputs (connected via thin dotted lines) the types of information required to inform a decision, which subsequently leads to actions taken that are defined as parameters. In this model, two key decisions are undertaken: decisions to allocate surveillance resources and decisions to apply mitigation measures, based on the outcome from surveillance. In this model, surveillance decisions are made on the basis of budgetary considerations, unit costs for different measures, the size of the population to be surveyed, and goals relating current disease incidence to actual incidence. As noted in Figure 3 and earlier in the paper, there will be feedbacks between the changing course of disease and the decision set for surveillance decisions that are incorporated in this model. Similarly, mitigation decisions are influenced by the type of surveillance undertaken, budget resources, mitigation costs, and detection efficacy, which in turn influence the course of disease (and, in the next period, the choice of surveillance strategy used). Thus, the operationalized model of Figure 4 incorporates the feedback structure found in Figure 3 concerning the relationships between surveillance, detection, mitigation, and control.

What about the overlays with socio-economic factors? In this case, the system dynamics model presented in Figure 4 can be used in conjunction with a variety of different economic frameworks. Following Rich [25], the epidemiology and economics could be linked through economic decisions that alter entry and exit rates (e.g., via births of animals, slaughter or breeding decisions) and transmission rates (via risk factors in production, rates of trade between regions, e.g.). Surveillance actions themselves can influence the economics if, for example, different strategies add costs for producers, which subsequently alter their production patterns in ways that could modulate the spread of disease as just described. 

The exact structure of the socio-economic side of the model will vary depending on context. Rich [25] utilized a population model of livestock that separated animals into different age cohorts that was linked with market demand for livestock products. As disease time steps in S-I-R models are often daily or weekly, such a population structure approach might be more amenable in species with shorter production cycles such as poultry. A more generic approach would be to directly model representative farm production decisions as a mathematical programming model in which typologies of agents maximize profits subject to various resource and other technical constraints. These types of models have been used in Scottish applications in recent years to look at the impact of animal health mitigations on farm- or herd-decisions [33,34]. In iThink, these models can be used to directly calculate the period-by-period optimization decisions of representative producers through an interface with Microsoft Excel. 

A disadvantage of using system dynamics models is that the level of aggregation of agents is often too broad. In an SD model, the system in question describes a representative system or agent (or average of agents), which may fail to capture the heterogeneity of farm types and actors in the system. An alternative in this case is to utilize an agent-based approach, where the agents involved can be individual-level farms or individuals, each with different types of rule systems that govern their behavior [35]. These rules can be based both on economic phenomenon (e.g., decisions to buy or hold cattle in response to changes market prices) and epidemiological ones (e.g., reactions to disease protocols). One of the advantages of such an approach is the possibility to encapsulate different aspects of the overall model, for example an on-farm disease model, a between-farm movements model, and a behavioural feedback model, with only a few well defined interactions between these modules. This means that the internal functionality of each module can then be extensively re-written without modifying the other modules. The main disadvantages of this approach are the computational requirements associated with such numerical methods, as well as the complexity in the underlying code and resultant difficulty in summarizing and evaluating the model. However, segregating the on-farm disease, movements, and behavioural feedback modules in this way greatly improves the legibility of the code for each aspect of the model while preserving all of the potential complexity of the full model, and fits nicely into an object-oriented programming framework.

We propose a framework for such an agent-based model written in the C++ programming language. At the core of this model is an object class representing a population of actors, with externally accessible methods to summarise and return the observed and latent state of each actor, as well as methods to create and remove actors from the population. This high-level object represents a single realisation of the system to be studied, and is given fixed parameters controlling the demography of the system such as the number and types of actors in the system, what the behavioural attributes of these actors are, the parameters governing test outcomes and disease transmission, and the economic model to be used. These parameters are used to set up multiple, possibly heterogenous lower level objects belonging to an actor class, each of which has external methods to return the observed and latent state of that actor (with internal rules determining the relationship between these states), methods to interact with other actors, and a set of internal rules defining how the behaviour of that actor is affected by these interactions. In the simplest case these actors need contain nothing else, although more complex farm classes inheriting from the parent class of actor will likely introduce complex on-farm disease dynamic models and diagnostic test representations. The actor class may also incorporate physical heterogeneity, such as herd size, farm type, breed of animals, proximity to water sources, as well as variations in the responses of farmers to external stimuli, using parameter values drawn from a distribution describing all actors at initialization of the class. The metapopulation object also contains regulator class objects representing the media and government influences on the system, which will be passed information on the observed disease state of the actors, and influence the behaviour of actors according to the media and government response to this observed disease state. Finally, the economic features of the system are controlled by a further regulator class, with interactions between this object and each actor further influencing the behaviour of these actors.

Each metapopulation object is allowed to run for a given time period and the required information is extracted from the system before another realisation of the class is invoked and allowed to run. In this way, a full distribution of possible outcomes given the same parameter set (representing variability in the system) is obtained. As the majority of parameters used to set up the simulation are likely to be at least partially unknown, the entire process can be repeated using multiple draws from probability distributions describing the uncertainty in these parameter estimates to determine the sensitivity of the system to the parameter values used.

Because of the inherent flexibility of our approach, this framework has a variety of potential uses in disease surveillance scenarios, as well as other applications in which behavioural influences between multiple actors need to be modelled. Therefore, it is our intention that this framework should be implemented in a generic and flexible way, and the development of new and modified classes to allow the framework to model new diseases and populations actively encouraged. 

## Results and Discussion

A simple illustration of the method was developed to assess the impact of farm heterogeneity and behavioural feedback effects on the observed prevalence of infected premises following a sudden increase in prevalence of farms positive for an endemic disease, for example due to change in climatic conditions favourable to the causative agent. The intention of this exemplar is to highlight the effects of behavioural feedback and heterogeneity in farm characteristics in terms of the number of animals, propensity to test for disease and on farm-disease prevalence. To facilitate interpretation of the results the true disease state of each farm is fixed and no epidemiological model for disease spread either within or between farms is incorporated.

For the first model, incorporating neither heterogeneity between farms nor behavioural feedback effects, a population of 1000 identical farms was established, with each farm possessing 100 animals and each with the same fixed diagnostic test sensitivity of 50 percent and specificity of 99.9 percent for an individual test on a single animal. At each time step, we assumed a 10 percent probability of testing a given farm for disease, with a 10 percent proportion of animals tested conditional on this, and untested farms preserving the disease classification held at the previous time point. To simulate the sudden increase in farm prevalence of disease, we began the simulation with 10 actors infected (i.e., 1 percent of farms), with a 20 percent on-farm prevalence of disease, and after 100 time steps an additional 90 farms were infected at a single time step, so that a total of 100 farms – 10 percent of the sample – were infected. The simulation was repeated 1000 times, recording the observed proportion of infected farms at each time step. 

This exercise was then repeated using a second model that incorporated heterogeneity between farms in terms of the number of animals on farm, the propensity to test for disease, and the proportion of animals tested – i.e., a situation that is vastly more likely to reflect reality than assuming all farms are identical. Note that although this is still likely to be simplified in relation to reality, the complexity of this between farm heterogeneity could be increased arbitrarily to include differences in farm type, risk factors such as spatial location or proximity to water, and differences in the responses of farmers to disease. The mean values for each parameter were taken from the corresponding statistics from the first model, but each farm was assigned a randomly generated value for each, with this parameter value then remaining fixed for each farm for that simulation but re-sampled between simulations. The test sensitivity and specificity remained fixed at 50 percent and 99.9 percent throughout. 

The third model returned to the assumption of no farm heterogeneity used for the first model, but incorporated the novel aspects of behavioural feedback discussed. Now, the baseline probability of each farm testing for disease and proportion of animals tested used for the first model were altered at each time step according to the output of an ‘Information’ regulator agent. This modulation was calculated in three parts: (a) an increase if the global prevalence had increased since the last time point and decrease if prevalence had fallen, (b) a linear effect of difference between the observed prevalence and a threshold of 7 percent prevalence, with observed prevalence higher than 7 percent resulting in an increase in propensity to test and vice versa, and the magnitude of the difference controlling the size of the effect, and (c) an increase for individual farms if that farm was classified as infected at the previous time point and decrease otherwise. At each time step, the overall feedback on propensity to test for disease was calculated and returned to each farm by the regulator before testing was simulated for that time step, to simulate the dynamic nature of these behavioural effects. Again, the complexity of these agents could be increased substantially, possibly incorporating elements of mandatory testing, rewards systems for disease freedom, or other forms of behaviour modification. 

Finally, a fourth model was constructed using both the heterogeneity and feedback mechanisms outlined above. For this model, the feedback of the Information regulator agent was applied to the farm specific baseline propensity to test for disease so that the heterogeneity between baseline farm attitudes to disease could be combined with the modulating effects of the observed disease prevalence, i.e. some farms will always look for disease harder than others, but all farms will look for disease more effectively if they believe they have a greater chance of having the disease. This is also complicated by the heterogeneity between farms in terms of number of animals, so that a farm with a larger number of animals is more likely to observe a positive test (either true or false positive) because of the larger number of tests applied.

**Figure 5:**
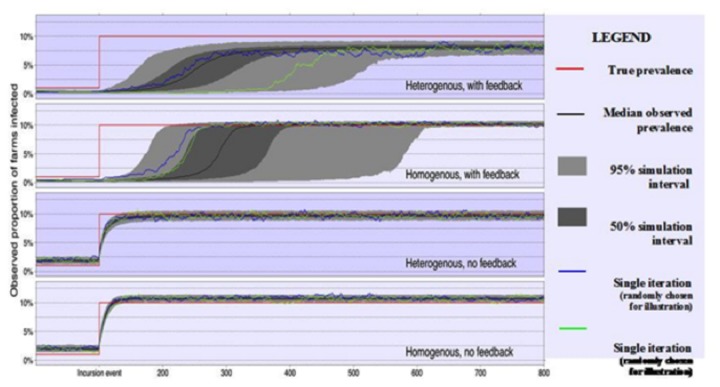
The effect of incorporating farm heterogeneity and/or behavioural feedback mechanisms on the observed prevalence of infected farms after a disease incursion event.

The results obtained from these models are shown in Figure 5. Incorporation of farm heterogeneity results in a reduction in the observed disease prevalence for these model parameters, due to the varying numbers of animals between farms and propensities to test for disease resulting in a greater number of false negative and a reduction in false positive results at a farm level. When behavioural feedback effects are incorporated into the model, we observed a greater increase in the time between disease incursion (i.e. at time 100) and the observed increase in disease prevalence due to the behavioural ‘lag time’ introduced by failing to detect disease in the initial stages when global prevalence is thought to be low and the disease is not perceived to be a threat. These two effects compound each other for model four, with the transition between observed low and high prevalence states prolonged for each individual simulation (examples shown in blue and green) by the sub-population of farms with small numbers of animals and a low propensity to test for disease, although the contrasting farms with higher propensity to test for disease do shorten the average lag phase between disease incursion and the initial increase in observed disease prevalence. Equally importantly, the stable observed disease prevalence for model four is considerably lower than each of the simpler models and the true figure of 10 percent. However, the magnitude and direction of these effects appear to be highly dependant on the parameter values used for both farm demography and behavioural effects (data not shown), and therefore not only reduce the precision of prevalence estimates, but also introduce a considerable source of bias. As can be seen from the example individual simulation outputs shown in blue and green on Figure 5, sequential prevalence estimates can be highly autocorrelated, which introduces a random-walk-like effect in the observed prevalence, when the true prevalence is in fact static. 

From these results, it is apparent that behavioural feedback mechanisms serve to reduce the usefulness of prevalence estimates based on diagnostic tests that are carried out at the behest of individual farms. However, mandatory surveillance, performed by a regulator and thus being independent of individual farm behavioural mechanisms, will produce prevalence estimates that are less dependent on these effects, which in turn may help to regulate the surveillance effort through a more representative overall prevalence estimate. Using an extension of the model incorporating both behavioural feedback and heterogeneity as discussed above, the impact of including different levels of mandatory surveillance on the number of voluntary surveillance diagnostic tests performed was assessed (Table 1). With the introduction of a small mandatory surveillance effort of ten farms, or even only a single farm per time step, the average number of passive surveillance tests performed per time step increased by substantially more than that introduced by the mandatory surveillance effort, which corresponded to a reduction in the average ‘lag time’ caused by failing to detect the increase in prevalence of disease in the early stages. This suggests that, at least for the parameter estimates used in our simulation, the majority of the value of mandatory surveillance may not be directly in the test results provided, but rather in the knock-on effect of behaviour modification increasing the propensity of farms to perform their own tests.

**Table 1:** The average number of farms performing passive surveillance and total passive tests performed per time step.

**Active surveillance effort**	**Passive tests done**	**Passive farms tested**	**Total tests done**	**Total farms tested**
100 farms	1220	91	2220	191
10 farms	879	73	979	83
1 farm	764	64	774	65
0 farms	676	58	676	58

Note: results obtained from an agent based simulation of behavioural feedback mechanisms with farm heterogeneity including active surveillance of 100, 10, 1 and 0 farms per time step, each with 10 tests per farm. Total tests done and farms tested incorporating both passive and active surveillance per time step also shown.

Although it is clear that there are some data deficient areas of potential importance in the framework presented, especially concerning behavioural aspects of farms, it is also clear that ignoring these effects on the basis that they are difficult to parametrise and assuming that no behavioural feedback mechanisms exist is likely to lead to misleading estimates for the effectiveness of disease surveillance programs. The framework presented is inherently flexible, and could be adapted to represent any given disease and surveillance system, as well as incorporating more detailed behavioural and economic feedback effects as required. The example shown here is deliberately simple, but illustrates how the approach could be used in more complex situations. Detailed simulation studies to quantify the sensitivity of the system to different estimates for key parameters would also be highly beneficial, and may identify key areas of knowledge deficit to be targeted in future studies of farm behaviour. 

## Conclusions

This paper has identified a number of important behavioral aspects involved in animal health surveillance systems, revealing that the effectiveness of surveillance protocols requires an appreciation of their systems context. The structure of a novel framework was presented that shows how estimates of prevalence can be significantly influenced by farmer behavior, with ramifications on the effectiveness of surveillance, disease spread, and resource allocation. The framework presented has a variety of potential uses in disease surveillance scenarios, as well as other applications in which behavioural influences may be important. 
